# 14-Deoxy-11,12-didehydroandrographolide Attenuates Lipotoxicity and Non-Alcoholic Steatohepatitis Through Restoration of Autophagy and Reduction in Oxidative Stress

**DOI:** 10.3390/ijms27177567

**Published:** 2026-08-24

**Authors:** Chia-Wen Lo, Yen-Chih Chen, Kai-Li Liu, Chien-Chun Li, Chong-Kuei Lii, Hsin-Hua Chan, Chih-Chieh Chen, Ya-Chen Yang, Haw-Wen Chen

**Affiliations:** 1 Department of Nutrition, College of Medical and Health Care, Hungkuang University, Taichung 433, Taiwan; cook0912@sunrise.hk.edu.tw; 2 Sports Medicine Division, Department of Orthopedics, Kuang Tien General Hospital, Taichung 433, Taiwan; orthft1040@ktgh.com.tw; 3 Department of Nutrition, Chung Shan Medical University, Taichung 402, Taiwan; kaililiu@csmu.edu.tw (K.-L.L.); licc@csmu.edu.tw (C.-C.L.); 4 Department of Nutrition, Chung Shan Medical University Hospital, Taichung 402, Taiwan; 5 Department of Nutrition, China Medical University, Taichung 406, Taiwan; cklii@mail.cmu.edu.tw (C.-K.L.); u110076203@cmu.edu.tw (H.-H.C.); 6 Department of Sports Medicine, China Medical University, Taichung 406, Taiwan; jason19821004@mail.cmu.edu.tw; 7 Department of Health and Nutrition Biotechnology, Asia University, Taichung 413, Taiwan; yachenyang@asia.edu.tw

**Keywords:** apoptosis, autophagy, 14-deoxy-11,12-didehydroandrographolide (deAND), lipotoxicity, non-alcoholic fatty liver disease (NAFLD), oxidative stress

## Abstract

Non-alcoholic fatty liver disease (NAFLD) is a prevalent metabolic disorder that can progress to non-alcoholic steatohepatitis (NASH), in which lipotoxicity, oxidative stress, apoptosis, and impaired autophagy contribute to liver injury. 14-Deoxy-11,12-didehydroandrographolide (deAND), a bioactive diterpenoid from Andrographis paniculata, has shown anti-inflammatory and antioxidant activities, but its role in NASH-associated lipotoxicity remains unclear. This study investigated the protective effects and underlying mechanisms of deAND using palmitic acid (PA)-treated AML12 hepatocytes and a choline-deficient, L-amino acid-defined, high-fat-diet (CDAHFD)-induced mouse model of NASH. In AML12 cells, PA impaired autophagic flux and reduced the expression of the mitophagy-associated proteins PINK1 and Parkin and increased p62, LC3-II, reactive oxygen species production, and apoptotic signaling. deAND treatment restored autophagic flux and increased PINK1 and Parkin expression, enhanced antioxidant defense-related proteins, including HO-1, GCLM, and GPX2, and reduced oxidative stress and apoptosis. The protective effects of deAND were attenuated by autophagy inhibitors, supporting the involvement of autophagy regulation. In CDAHFD-fed mice, deAND reduced hepatic steatosis, inflammation, fibrosis, apoptosis, and autophagy dysregulation. These findings suggest that deAND alleviates lipotoxic liver injury by restoring autophagic homeostasis and reducing oxidative stress.

## 1. Introduction

Non-alcoholic fatty liver disease (NAFLD) is a chronic metabolic condition defined by hepatic lipid accumulation exceeding 5% of liver weight in individuals with negligible alcohol consumption [1]. Currently affecting approximately 25% of the global population, NAFLD is frequently comorbid with type 2 diabetes, cardiovascular disease, and chronic kidney disease [2]. Beyond its clinical impact on patient quality of life, the disease places a substantial economic burden on global healthcare systems, making its prevention and management a critical public health priority [3]. The progression of NAFLD typically begins with simple steatosis (NAFL) but can advance to non-alcoholic steatohepatitis (NASH), a more severe stage marked by lobular inflammation, macrophage infiltration, hepatocyte ballooning, and fibrosis. Without intervention, NASH may culminate in cirrhosis or hepatocellular carcinoma (HCC) [4]. The pathogenesis is inherently multifactorial, partly driven by elevated circulating free fatty acids (FFAs) secondary to dietary habits or metabolic impairment. These elevated FFAs serve as a central trigger, compromising insulin sensitivity and gut microbiota homeostasis while simultaneously inducing oxidative stress and systemic cellular damage, a toxic response named lipotoxicity [5,6]. These interconnected factors create a self-perpetuating cycle that accelerates liver injury [7].

Lipotoxicity describes the deleterious accumulation of lipid species in non-adipose tissues like the liver, resulting in profound cellular dysfunction [8]. This occurs when the balance between lipogenesis, beta-oxidation, and lipid export is disrupted [9]. Lipotoxic mediators, notably palmitic acid (PA), ceramides, and diacylglycerols, act as primary triggers for endoplasmic reticulum (ER) stress and mitochondrial dysfunction. The consequent surge in reactive oxygen species (ROS) serves as a critical driver, propelling the progression of hepatic inflammation and subsequent fibrogenesis [8].

A key mechanism in maintaining cellular homeostasis is autophagy, a catabolic process that removes damaged organelles and unnecessary components. In the context of NAFLD, dysregulated autophagy impairs hepatic function and triggers apoptosis [10]. Chronic FFA exposure disrupts mitochondrial integrity and suppresses mitophagy [11]. Clinically, NASH patients often exhibit elevated p62 levels and increased LC3-II/LC3-I ratios alongside proapoptotic markers like caspase-3, signaling a blockade in autophagic flux [12]. Research suggests that while deficient autophagy exacerbates lipid retention, the restoration of autophagic flux can significantly attenuate hepatic steatosis [13].

Andrographis paniculata is a traditional Asian medicinal herb used to treat various inflammatory and metabolic ailments [14]. While its primary constituent, andrographolide (AND), is well documented for its antioxidant and anti-inflammatory properties [15,16], its analogue, 14-deoxy-11,12-didehydroandrographolide (deAND), has emerged as a promising candidate due to its lower cytotoxicity and superior bioavailability [15,17]. Recent studies indicate that deAND possesses potent hepatoprotective effects such as inhibiting the NLRP3 inflammasome and activating Nrf2-mediated antioxidant defenses [18].

Despite these preliminary findings, the specific role of deAND in modulating autophagic pathways during NASH progression remains largely unexplored. In this study, we employed palmitic acid-induced AML12 hepatocytes and a choline-deficient, L-amino acid-defined, high-fat-diet (CDAHFD)-fed mouse model to investigate the therapeutic potential of deAND, focusing on its ability to mitigate lipotoxicity by restoring autophagic flux and reducing oxidative stress and apoptosis.

## 2. Results

### 2.1. Effects of PA and deAND on Cell Viability in AML12 Cells

**Figure 1:**
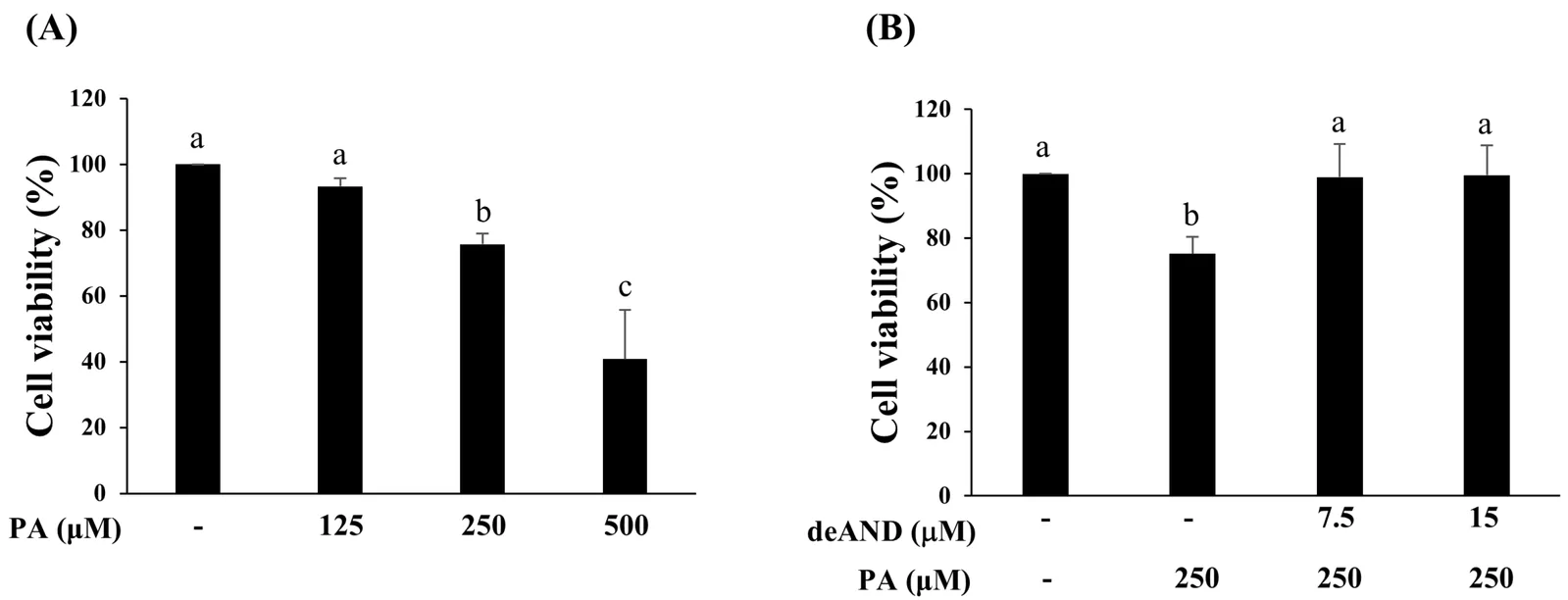
Effects of PA and deAND on AML12 cell viability. (**A**) AML12 cells were exposed to increasing concentrations of PA (125, 250, or 500 μM) for 24 h. (**B**) Cells were pretreated with deAND (7.5 or 15 μM) for 16 h prior to incubation with 250 μM of PA for an additional 24 h. Data are presented as mean ± SD from three independent experiments. Groups labeled with different letters indicate statistically significant differences (*p* < 0.05).

The impact of PA on AML12 cell viability was assessed in a dose-dependent manner using the MTT assay. Cells were exposed to 125, 250, or 500 μM of PA for 24 h. As illustrated in Figure 1A, treatment with 250 and 500 μM of PA led to a significant, concentration-dependent decrease in cell viability. To evaluate the protective role of deAND, cells were pretreated with 7.5 or 15 μM of deAND for 16 h, followed by incubation with 250 μM of PA for an additional 24 h. As shown in Figure 1B, pretreatment with deAND markedly alleviated the reduction in cell viability caused by PA. Overall, these results suggest that PA at concentrations of 250 μM or higher significantly impairs cell viability, while deAND pretreatment effectively reduces this cytotoxic effect.

### 2.2. PA Induces Apoptosis While Suppressing Autophagic Flux and PINK1/Parkin-Associated Mitophagy Signaling in AML12 Cells

**Figure 2:**
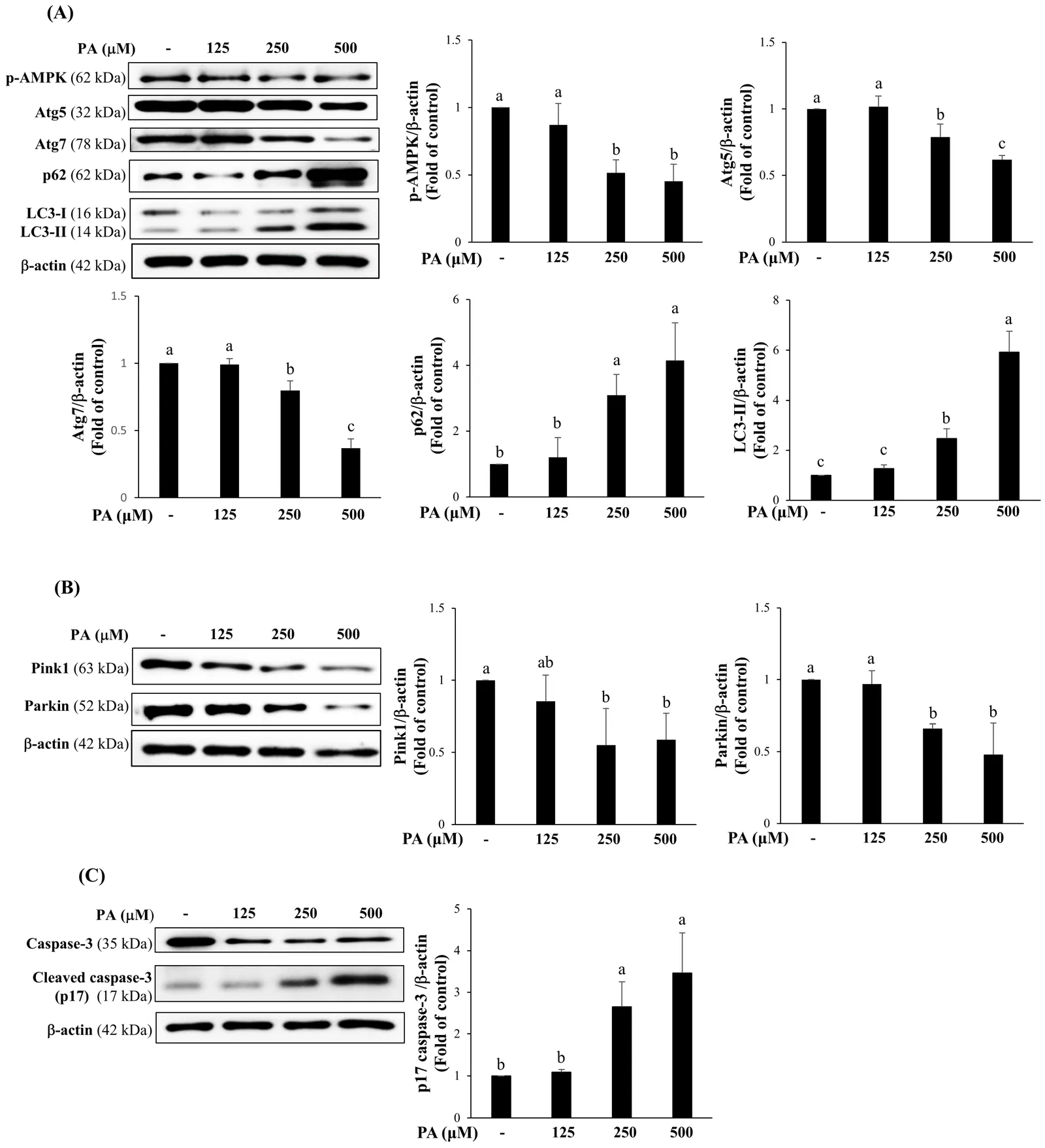

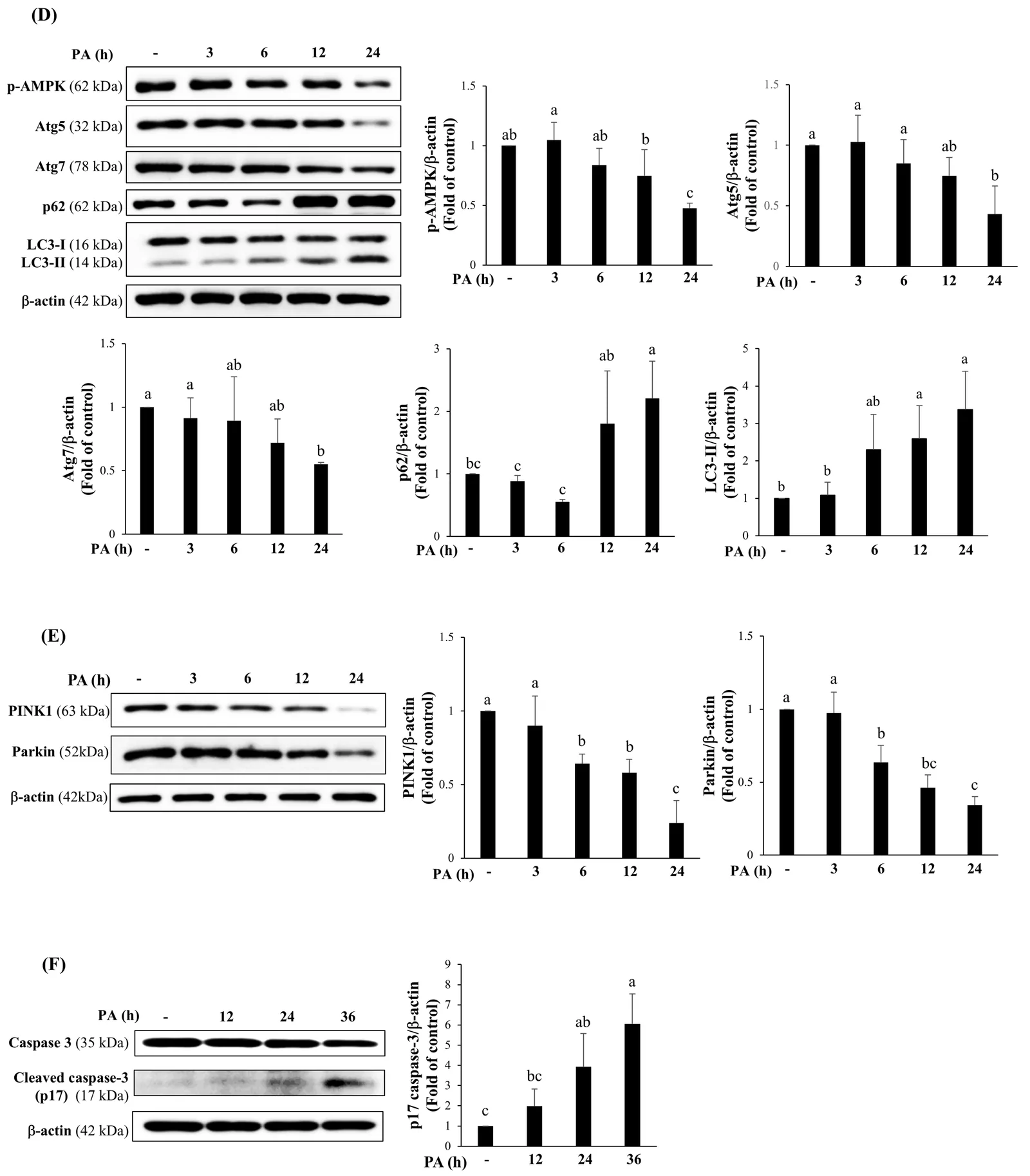
PA induces apoptosis while inhibiting autophagic flux and PINK1/Parkin-associated mitophagy signaling in AML12 cells. (**A**–**C**) Cells were treated with increasing concentrations of PA (125, 250, or 500 μM) for 24 h. (**D**,**E**) Cells were exposed to 250 μM of PA for different time periods (3, 6, 12, or 24 h). (**F**) Cells were treated with 250 μM of PA for 12, 24, or 36 h. Data are presented as mean ± SD from three independent experiments. Different letters indicate statistically significant differences between groups (*p* < 0.05).

To examine the dose-dependent effects of PA on autophagic flux, PINK1/Parkin-associated mitophagy signaling, and apoptosis, AML12 cells were treated with various concentrations of PA (0–500 μM) for 24 h. As shown in Figure 2A, PA exposure resulted in a dose-dependent reduction in the expression of autophagy-associated proteins, including phosphorylated AMPK (p-AMPK), Atg5, and Atg7. In contrast, total p62 and LC3-II levels increased significantly with higher PA concentrations, suggesting impaired autophagic degradation and blockade of autophagic flux. Furthermore, the expression of mitophagy-related proteins PINK1 and Parkin was decreased in a dose-dependent manner (Figure 2B). PA treatment also elevated the levels of p17 caspase-3, indicating enhanced apoptosis (Figure 2C). To further assess the time-dependent effects, AML12 cells were treated with 250 μM of PA for different durations. As illustrated in Figure 2D, the expression of p-AMPK, Atg5, and Atg7 progressively declined over time, with significant reductions observed at 24 h. Conversely, total p62 and LC3-II levels increased markedly after 12 and 24 h, further supporting the suppression of autophagic flux over time. Consistently, PINK1/Parkin-associated mitophagy signaling was progressively reduced, as indicated by decreased PINK1 and Parkin expression (Figure 2E). Meanwhile, cleaved caspase-3 levels increased after 24 h of PA exposure and continued to rise up to 36 h, confirming the induction of apoptosis (Figure 2F). Collectively, these results indicate that PA disrupts autophagic flux and suppresses PINK1/Parkin-associated mitophagy signaling while promoting apoptosis in AML12 cells.

### 2.3. deAND Restores PA-Impaired Autophagic Flux, Modulates PINK1/Parkin-Associated Mitophagy Signaling, and Attenuates Apoptosis in AML12 Cells

**Figure 3:**
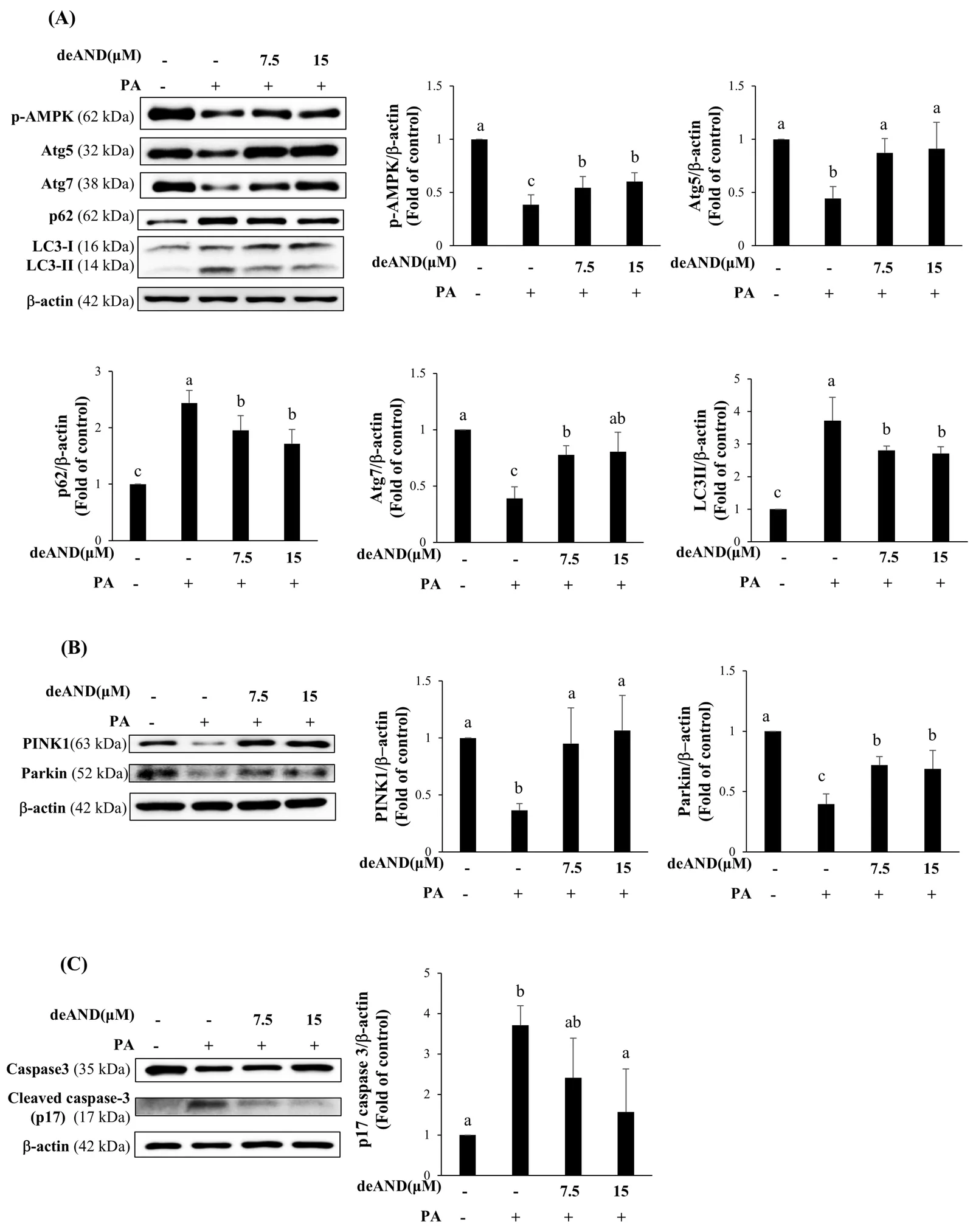
deAND alleviates PA-induced apoptosis and restores suppressed autophagy and PINK1/Parkin-associated mitophagy signaling in AML12 cells. Cells were pretreated with deAND (7.5 or 15 μM) for 16 h prior to exposure to 250 μM of PA. (**A**,**B**) Autophagy- and mitophagy-related protein expression was evaluated after 24 h of PA treatment, (**C**) whereas apoptosis-related protein levels were assessed after 36 h. Data are presented as mean ± SD from three independent experiments. Groups not sharing the same letter indicate statistically significant differences (*p* < 0.05).

To determine whether deAND could alleviate the cytotoxic effects of PA in AML12 cells, cells were pretreated with deAND prior to PA exposure. As shown in Figure 3A, deAND pretreatment significantly restored the levels of autophagy-related proteins, including p-AMPK, Atg5, and Atg7, which were reduced by PA. Moreover, deAND markedly decreased the PA-induced accumulation of p62 and LC3-II. deAND pretreatment also reversed the PA-mediated downregulation of PINK1 and Parkin, as illustrated in Figure 3B. In addition, deAND significantly suppressed the PA-induced increase in p17 caspase-3 levels (Figure 3C). Collectively, these findings indicate that deAND pretreatment mitigates PA-induced defects in autophagic flux and PINK1/Parkin-associated mitophagy signaling and reduces apoptotic signaling in AML12 cells.

### 2.4. deAND Reduces PA-Induced Autophagosome Accumulation and Apoptosis in AML12 Cells

**Figure 4:**
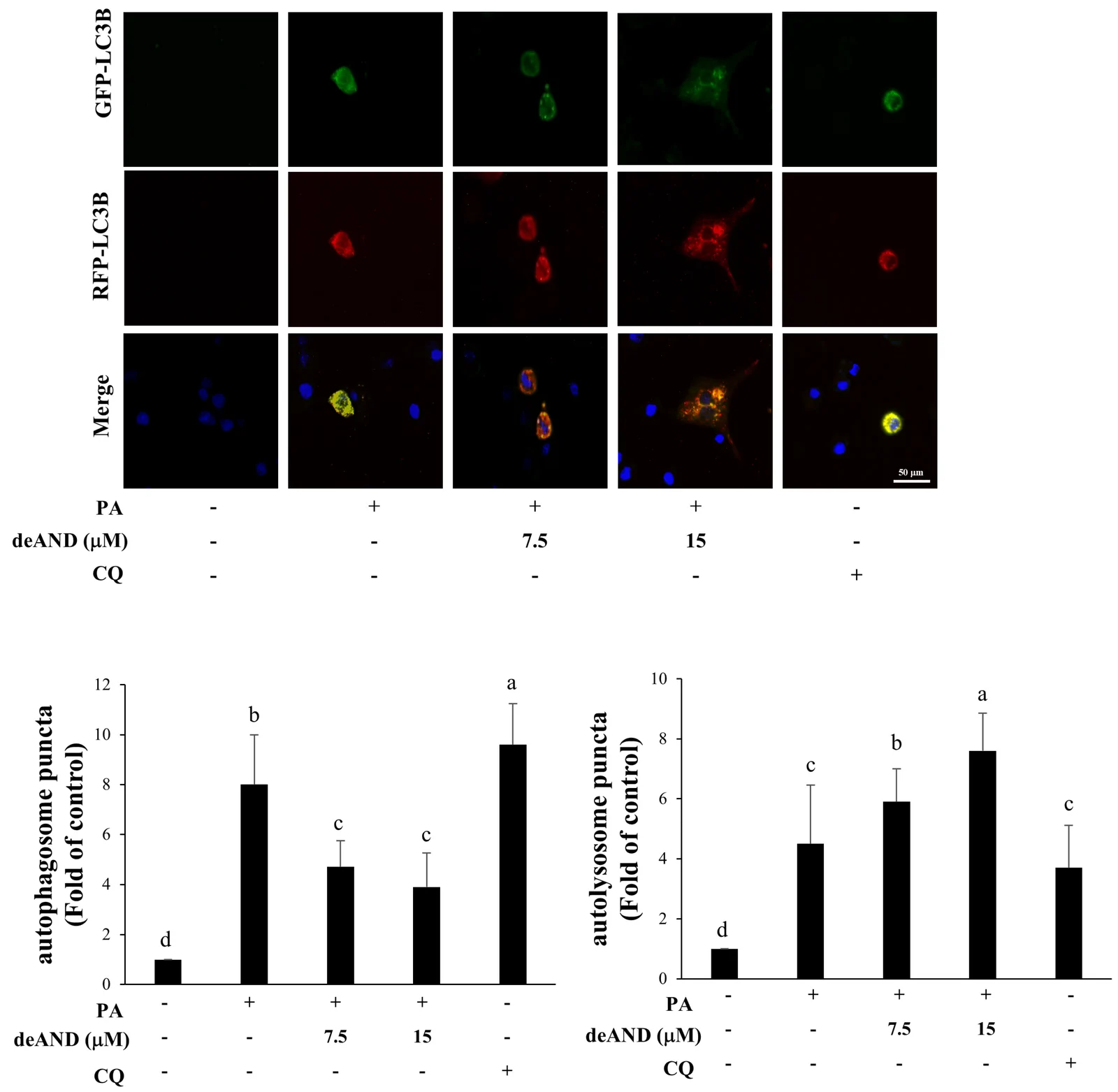
deAND promotes autophagic flux in AML12 cells. AML12 cells were transfected with an RFP-GFP-LC3B plasmid for 24 h and then exposed to deAND (7.5 or 15 μM) for 16 h, followed by treatment with 250 μM of palmitic acid (PA) for 8 h. Cells treated with 30 μM of chloroquine (CQ) were used as a late-stage autophagic flux inhibitor. Green fluorescence represents GFP-LC3B, red fluorescence represents RFP-LC3B, and blue fluorescence represents DAPI-stained nuclei. In the merged images, yellow puncta (GFP^+^/RFP^+^) indicate autophagosomes, whereas red-only puncta (GFP^−^/RFP^+^) indicate autolysosomes. Images were acquired at 400× magnification. Data are presented as mean ± SD from five independent experiments. Different letters indicate statistically significant differences (*p* < 0.05).

The proteins p62 and LC3-II are commonly used indicators of autophagic activity [19]. Although autophagosome formation reflects the initiation of autophagy, effective autophagic function depends on the completion of autophagic flux, defined by the degradation of cellular components following autophagosome–lysosome fusion. To determine whether deAND influences autophagic flux in PA-treated AML12 cells, autophagosome and autolysosome formation were assessed using a tandem fluorescent-tagged LC3B reporter. As shown in Figure 4, PA treatment significantly increased autophagosome accumulation compared to the control group (*p* < 0.05), indicating disruption of autophagic flux. Importantly, pretreatment with deAND reduced autophagosome numbers while increasing autolysosome formation, suggesting a restoration of autophagic flux.

**Figure 5:**
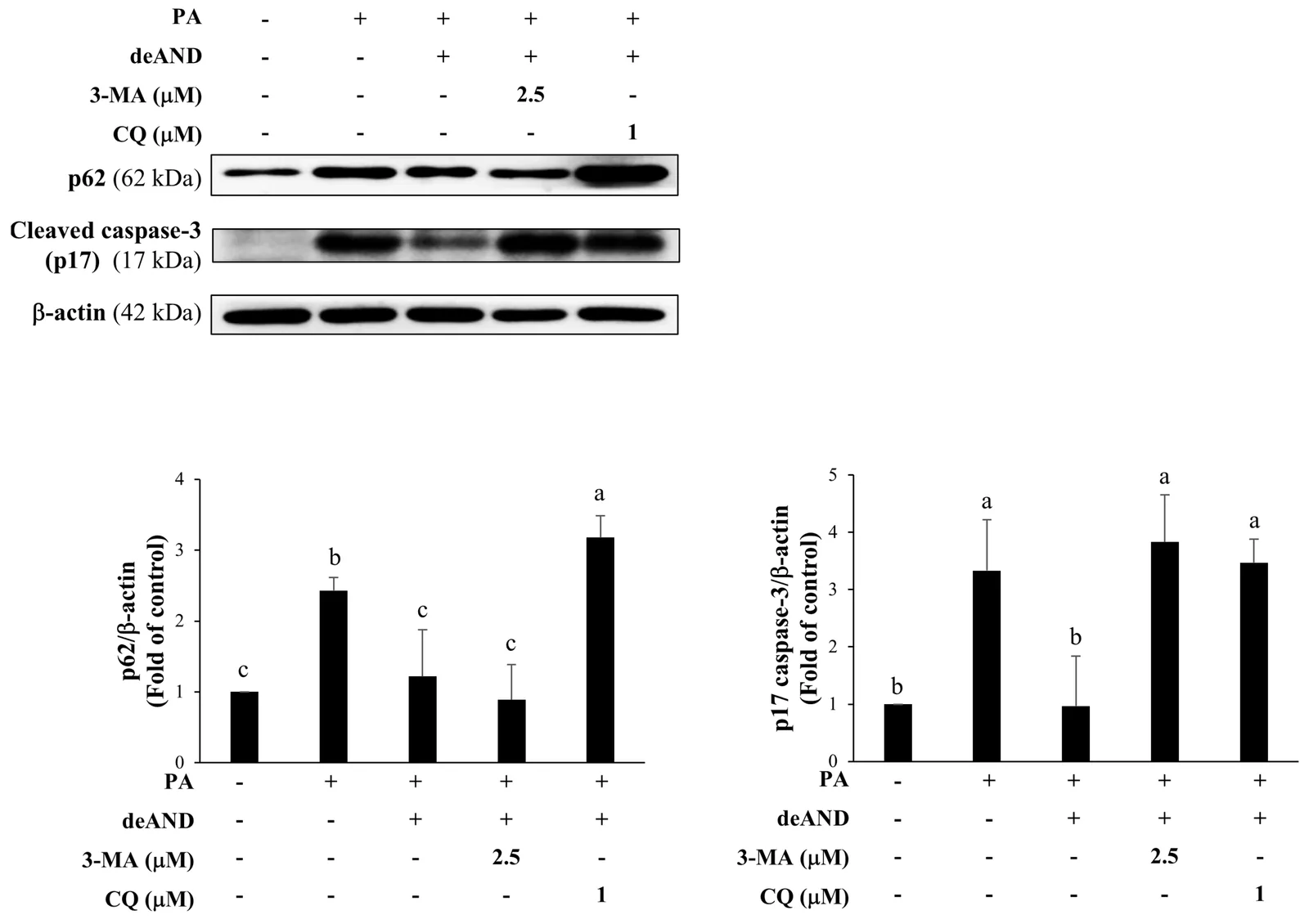
deAND attenuates palmitic acid (PA)-induced autophagosome accumulation and apoptosis in AML12 cells. Cells were pretreated with 15 μM of deAND for 16 h, followed by exposure to either 2.5 mM of 3-MA or 1 mM of CQ for 3 h. The cells were then treated with 250 μM of PA for an additional 24 h. Data are presented as mean ± SD from five independent experiments. Different letters indicate statistically significant differences (*p* < 0.05).

To further clarify the involvement of autophagy in deAND-mediated prevention, two autophagy inhibitors were used: 3-methyladenine (3-MA), which suppresses autophagy initiation by inhibiting class III PI3K activity, and chloroquine (CQ), which blocks late-stage autophagic flux by impairing lysosomal acidification and autophagosome–lysosome degradation. PA treatment markedly increased the levels of p62 and p17 caspase-3, indicating impaired autophagic flux and enhanced apoptosis (Figure 5). Pretreatment with deAND reduced both p62 accumulation and p17 caspase-3 expression, suggesting that deAND alleviates PA-induced autophagic dysfunction and apoptotic activity. However, co-treatment with 3-MA abolished the inhibitory effect of deAND on p17 caspase-3 and was accompanied by a further decrease in p62 levels. Because p62 abundance is regulated by multiple processes, this decrease cannot be interpreted solely as a direct measure of autophagic flux. In contrast, CQ co-treatment markedly increased p62 accumulation and p17 caspase-3 expression, consistent with impaired lysosomal degradation and enhanced apoptotic signaling. Taken together, these findings support an association between intact autophagic function and the antiapoptotic effects of deAND, although potential autophagy-independent effects of 3-MA and CQ cannot be excluded.

### 2.5. deAND Suppresses PA-Induced ROS Generation in AML12 Cells

**Figure 6:**
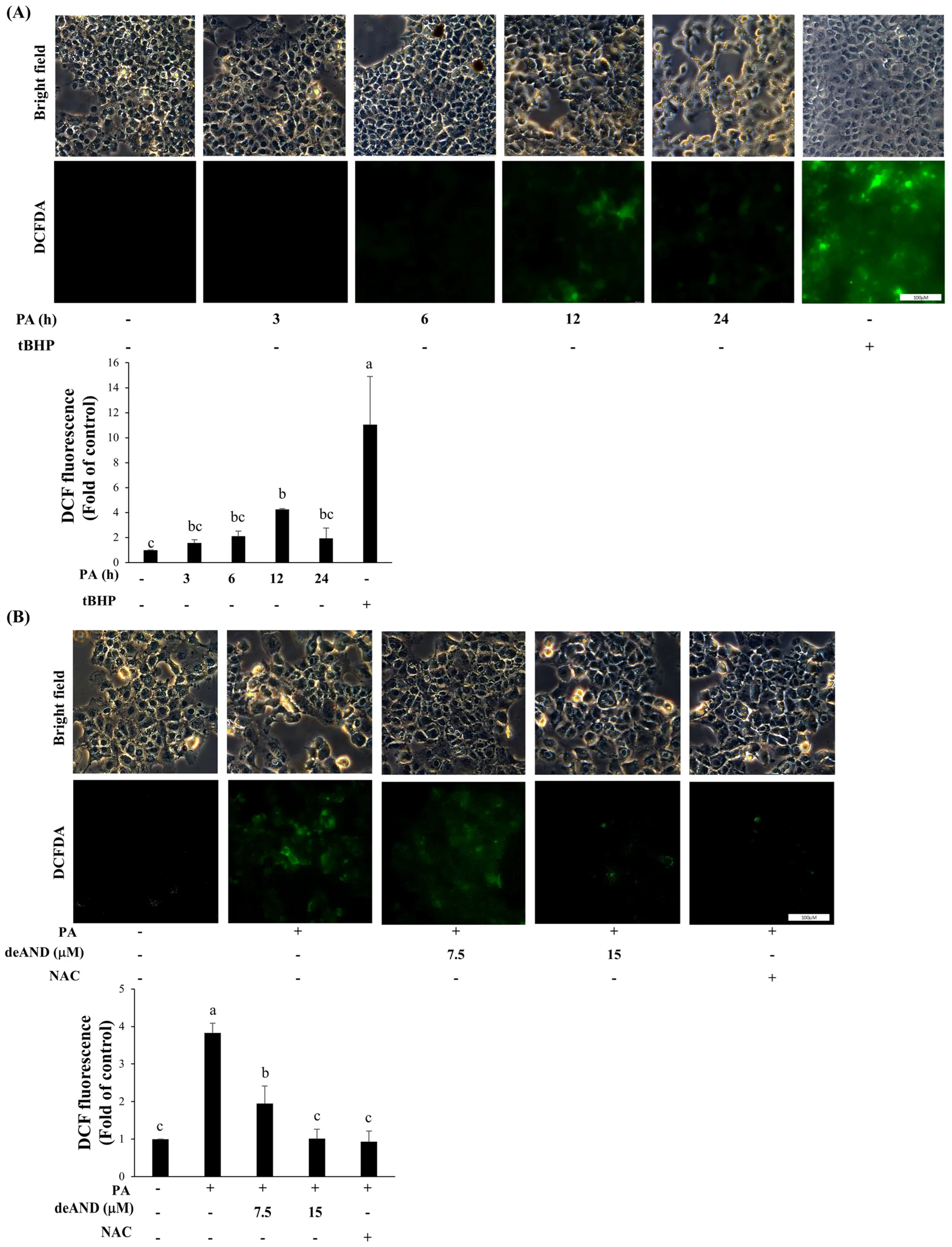

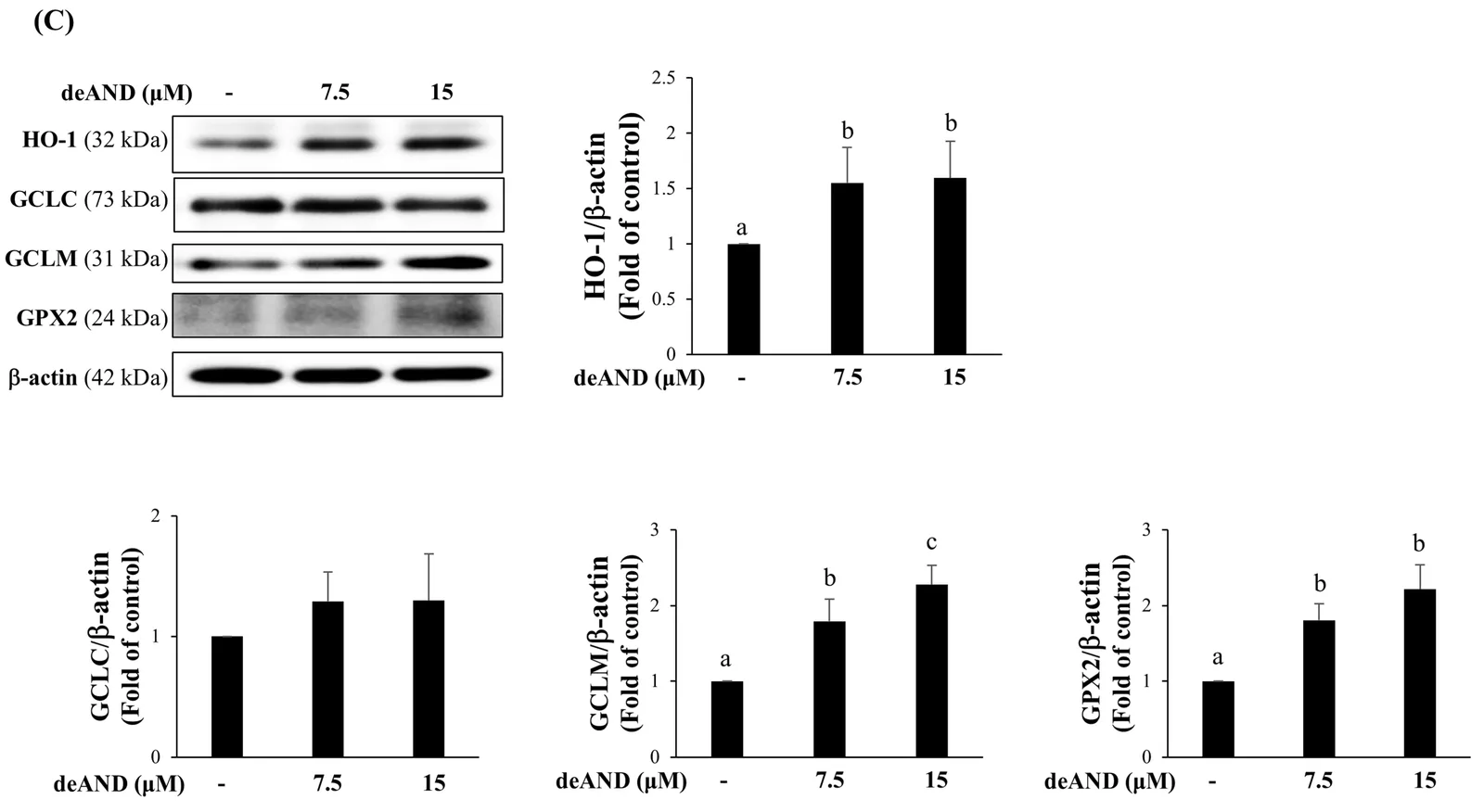
deAND inhibits PA-induced ROS generation and enhances antioxidant enzyme expression in AML12 cells. (**A**) Cells were treated with 250 μM of PA for 3, 6, 12, or 24 h or with 250 μM of tBHP for 30 min. tert-Butyl hydroperoxide (tBHP) served as a positive control for ROS generation. (**B**) Cells were pretreated with deAND (7.5 or 15 μM) for 16 h or NAC (5 mM) for 4 h, followed by exposure to 250 μM of PA for 12 h. NAC was used as a positive control for ROS inhibition. Intracellular ROS levels were measured using DCFDA staining and fluorescence microscopy. (**C**) Cells were treated with deAND (7.5 or 15 μM) for 16 h to assess antioxidant enzyme expression. Images were acquired at 200× magnification. Data are expressed as mean ± SD from three independent experiments. Different letters indicate statistically significant differences (*p* < 0.05).

PA-induced apoptosis has been strongly associated with excessive production of reactive oxygen species (ROS) [20]. deAND is known to exhibit antioxidant properties by upregulating enzymes such as glutathione peroxidase (GPX) and heme oxygenase-1 (HO-1) [18]. To determine whether the deAND-mediated enhancement of autophagic flux and reduction in apoptosis are linked to its antioxidant activity, we first evaluated the time-dependent generation of ROS in AML12 cells following PA exposure. As shown in Figure 6A, ROS levels reached their peak at 12 h after PA treatment, and this time point was used for subsequent experiments. As illustrated in Figure 6B, pretreatment with 7.5 or 15 μM of deAND significantly reduced ROS production induced by 250 μM of PA. N-acetylcysteine (NAC), a ROS scavenger, was used as a positive antioxidant control.

We further examined whether deAND influences the expression of key antioxidant enzymes. As shown in Figure 6C, deAND increased the protein levels of HO-1, GCLM, and GPX2, while having no significant effect on GCLC.

Overall, these results indicate that deAND mitigates PA-induced oxidative stress in AML12 cells, likely through the upregulation of antioxidant defense-related proteins, including HO-1, GCLM, and GPX2.

### 2.6. deAND Attenuates CDAHFD-Induced Steatohepatitis and Liver Fibrosis

**Figure 7:**
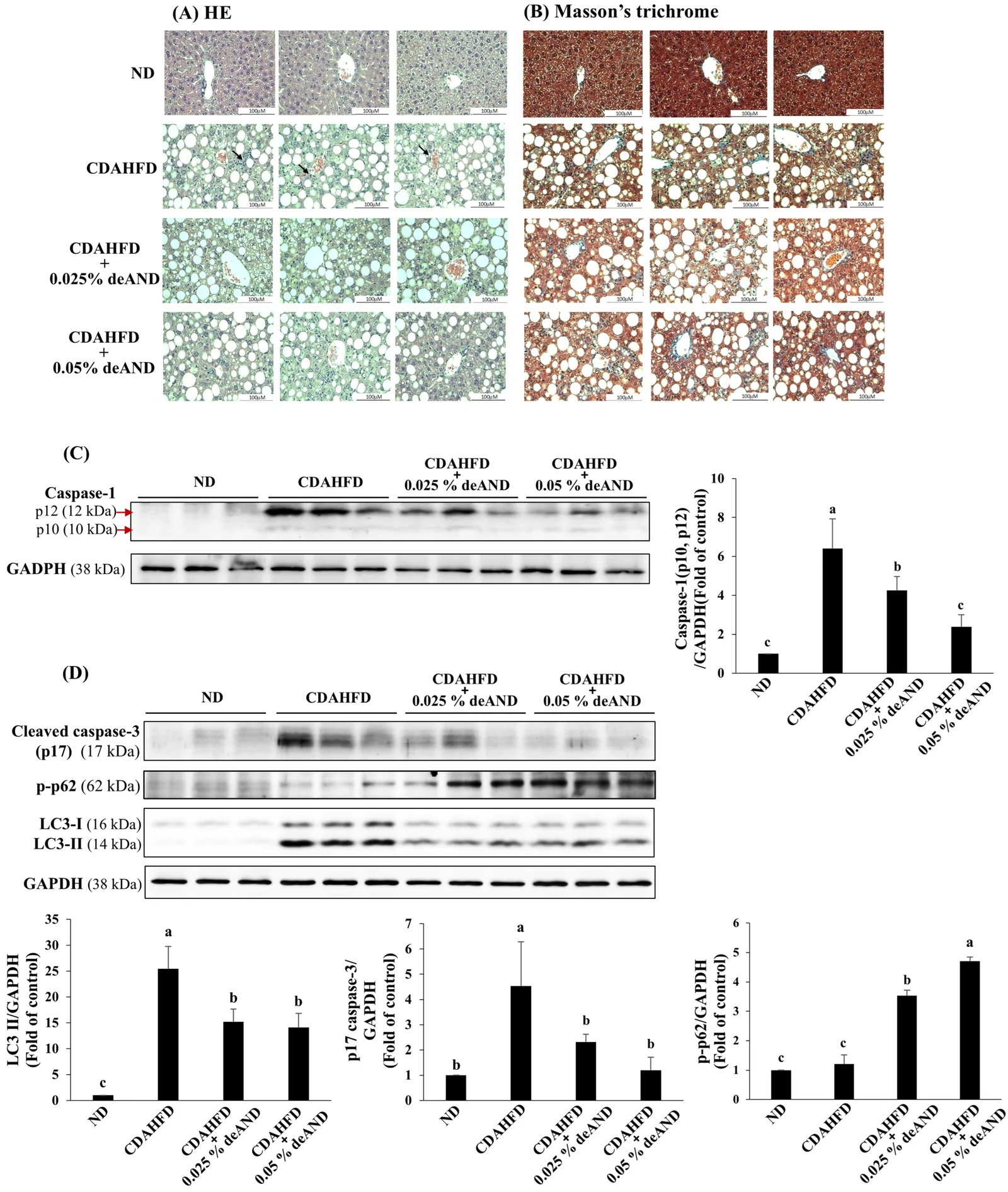
deAND attenuates CDAHFD-induced NASH. Mice were fed a choline-deficient, L-amino acid-defined, high-fat diet (CDAHFD) for 6 weeks to induce nonalcoholic steatohepatitis (NASH), with concurrent administration of 0.025% or 0.05% deAND to evaluate its protective effects. (**A**) Hepatic lipid accumulation and inflammation were determined by H&E staining. Black arrows indicate periportal inflammatory cell infiltration. (**B**) Liver fibrosis was determined by Masson’s staining. (**C**) Protein expression of inflammation-related markers in liver tissue. (**D**) Expression levels of autophagy-related and apoptosis-related proteins in liver tissues. Data are expressed as mean ± SD from three independent experiments. Different letters indicate statistically significant differences (*p* < 0.05). H&E staining was used to evaluate hepatic steatosis and inflammatory cell infiltration, whereas Masson’s trichrome staining was used to assess collagen deposition and fibrosis. Images were acquired at 400× magnification. A standardized histological scoring system based on the NAFLD activity score (NAS) was not applied.

Mice were fed a CDAHFD for 6 weeks to induce steatohepatitis, while receiving either 0.025% or 0.05% deAND to assess its ameliorating effects. As shown in Figure 7A, CDAHFD feeding markedly increased hepatic lipid accumulation and inflammation. In contrast, co-administration of deAND alleviated steatosis and inflammatory responses, as indicated by reduced inflammatory cell infiltration. Consistent with these histological changes, serum aspartate aminotransferase (AST; historically referred to as GOT), a biochemical indicator of liver injury, was markedly elevated in the CDAHFD group compared with the ND group (286.8 ± 17.9 vs. 73.4 ± 15.6 U/L, *p* < 0.05). Notably, co-treatment with 0.025% and 0.05% deAND significantly attenuated this increase, reducing AST levels to 222.6 ± 14.4 and 214.4 ± 14.9 U/L, respectively (*p* < 0.05 vs. CDAHFD). In addition to steatosis and inflammation, CDAHFD also markedly promoted liver fibrosis, as evidenced by Masson’s trichrome staining (Figure 7B), whereas deAND treatment substantially attenuated this fibrotic change. Furthermore, beyond macrophage infiltration, caspase-1 (p12) protein expression, an indicator of inflammasome-associated caspase-1 activation and a key mediator of IL-1β production, was markedly elevated in the CDAHFD group relative to the normal diet (ND) group. deAND treatment dose-dependently suppressed this increase (Figure 7C).

Regarding autophagy, CDAHFD significantly increased LC3-II protein levels compared with the ND group, whereas co-treatment with deAND attenuated this increase (Figure 7D). Furthermore, CDAHFD induced p62 phosphorylation, which was further augmented by deAND treatment, potentially reflecting enhanced cargo recognition during selective autophagy (Figure 7D). CDAHFD also markedly elevated the protein expression of p17 caspase-3, while deAND co-treatment dose-dependently suppressed this induction (Figure 7D).

Collectively, these results demonstrate that CDAHFD induces hepatic steatosis, inflammation, fibrosis, and apoptosis, whereas deAND attenuates these pathological alterations. The hepatoprotective effects of deAND were accompanied by changes in autophagy-associated markers, although the mechanistic role of autophagy in vivo remains to be fully established.

## 3. Discussion

As global obesity rates continue to increase, the prevalence of NAFLD has risen accordingly [3]. Lipotoxicity, commonly associated with dietary factors or metabolic disorders, is characterized by elevated circulating FFAs. These FFAs, particularly PA, accumulate in non-adipose tissues such as the liver and contribute to hepatocellular injury [8]. Emerging evidence indicates that liver damage is not primarily caused by total hepatic triglyceride content but, rather, by PA and other lipotoxic intermediates such as ceramides, diacylglycerols, and lysophosphatidylcholines [8]. Among these, PA has been extensively reported to induce hepatotoxicity [21,22]. Therefore, PA was used in this study to establish a lipotoxicity model in AML12 mouse hepatocytes and to evaluate the preventive effects of deAND. Furthermore, a CDAHFD-based NASH model, modified from the methionine- and choline-deficient diet, was utilized in the in vivo study owing to its established capacity to induce hepatic steatosis by disrupting triglyceride secretion, subsequently promoting steatohepatitis and fibrosis [23]. Compared with the methionine- and choline-deficient diet, the CDAHFD contains a moderately reduced methionine level, which prevents significant weight loss and partially recapitulates certain pathological features of human NASH [24]. Additionally, the CDAHFD model offers practical advantages, including a shorter experimental duration and lower cost [24]. Our findings demonstrate that deAND effectively attenuated lipotoxicity-associated steatohepatitis, hepatic injury, and fibrotic progression in both PA-treated AML12 hepatocytes and CDAHFD-fed mice. Consistent with the histological improvement in hepatic steatosis observed in the present CDAHFD model, our previous study using an HFHC diet-induced steatohepatitis model demonstrated that deAND reduced hepatic lipid accumulation and ameliorated hepatic steatosis [18]. These findings support an effect of deAND on hepatic lipid accumulation, although cholesterol specifically associated with isolated hepatic lipid droplets was not quantified in the present study.

Beyond lipid accumulation, disruptions in endoplasmic reticulum function, autophagy, and mitochondrial activity play central roles in NASH progression [25,26]. Impaired autophagic flux, mitochondrial dysfunction, and increased apoptosis have been observed in both human NASH and rodent NAFLD models [12,27,28]. In the present study, 24 h PA exposure reduced the expression of autophagy-related proteins (p-AMPK, Atg5, and Atg7) and mitophagy markers (PINK1 and Parkin), while increasing markers of impaired autophagy (p62 and LC3-II) and apoptosis (p17 caspase-3). These findings indicate that prolonged PA exposure is associated with impaired autophagic flux, reduced PINK1/Parkin-associated mitophagy signaling, and increased apoptosis. Because mitophagic flux was not directly assessed using a mitophagy-specific reporter or mitochondrial–lysosomal colocalization, changes in PINK1 and Parkin expression should be interpreted as modulation of mitophagy-associated signaling rather than direct evidence of mitophagic flux. Autophagy induction relies heavily on the AMPK-ULK1 signaling pathway; consequently, diminished AMPK activity can impair the initiation of this process. It is well established that while AMPK stimulates ULK1-dependent autophagy, mechanistic target of rapamycin complex 1 (mTORC1) acts to suppress it [29]. Because the cargo receptor p62/SQSTM1 is selectively cleared by autophagy, its accumulation serves as an indicator of defective autophagic degradation. Additionally, p62 can further disrupt autophagy signaling due to its scaffolding role in driving mTORC1 activation [30]. Collectively, the changes in p-AMPK, Atg5, Atg7, p62, and LC3-II are consistent with the involvement of AMPK-associated autophagy regulation in PA-induced autophagic dysfunction. However, because AMPK inhibition or genetic manipulation was not performed, the present findings do not establish AMPK activation as a causal upstream mediator of the effects of deAND. Notably, consistent with the reduction in apoptosis observed following deAND treatment, the PA-induced alterations in autophagy-related proteins were markedly attenuated. Specifically, deAND reversed the suppression of p-AMPK, Atg5, and Atg7 and restored the expression of the mitophagy-associated proteins PINK1 and Parkin, while mitigating the accumulation of p62 and LC3-II in hepatocytes. These findings suggest that the preventive effects of deAND are attributable, at least in part, to its ability to preserve autophagic homeostasis and prevent autophagy dysregulation in response to lipotoxic stress.

Impaired autophagic flux is increasingly recognized as a hallmark of NAFLD and a driving factor in NASH progression [27]. In animal models, high-fat-diet-induced NASH is associated with autophagosome accumulation [27]. Although LC3-II accumulation has traditionally been interpreted as increased autophagy, it may also indicate defective degradation [31]. Therefore, assessment of autophagic flux is more informative than static LC3-II levels. In this study, both Western blot analysis and tandem RFP-GFP-LC3B fluorescence imaging demonstrated that PA increased autophagosome formation without efficient degradation, whereas deAND enhanced autolysosome formation, indicating restoration of autophagic flux.

Given the close relationship between autophagy dysfunction and apoptosis in NASH development [32], we further investigated their interplay. Treatment with the autophagy inhibitors 3-MA (an inhibitor of autophagy initiation) and chloroquine (CQ; an inhibitor of autophagic degradation) demonstrated that PA increased the levels of both p62 and p17 caspase-3. Pretreatment with deAND attenuated these increases. Nevertheless, the antiapoptotic effect of deAND was eliminated by either 3-MA or CQ. In contrast, deAND-mediated suppression of p62 accumulation was abolished by CQ but not by 3-MA. These findings highlight the distinct actions of these inhibitors and support the notion that intact autophagic flux is essential for the antiapoptotic effects of deAND. However, both 3-MA and CQ have autophagy-independent effects, and inhibitor-alone controls were not included in the present study. Therefore, these findings should be interpreted cautiously. In addition, the decrease in p62 observed with 3-MA highlights that p62 abundance reflects multiple regulatory processes and should not be considered a direct measure of autophagic flux in isolation.

In addition to the in vitro experiments, an in vivo study using mice fed a CDAHFD was conducted to evaluate the ameliorating effects of deAND against CDAHFD-induced NASH, liver fibrosis, and apoptosis, as well as its potential role in regulating autophagy. CDAHFD feeding successfully induced hepatic steatosis, inflammation, fibrosis, and apoptosis, all of which were attenuated by deAND co-treatment. LC3-II was increased in CDAHFD-fed mouse livers, indicating altered autophagy regulation or autophagosome accumulation under hepatic stress. deAND reduced LC3-II accumulation, suggesting attenuation of CDAHFD-induced autophagy dysregulation. Furthermore, phosphorylation of p62 at Ser403, which enhances its binding to ubiquitinated proteins and facilitates their degradation via autophagy [33], was increased by CDAHFD and further enhanced by deAND. This finding suggests that deAND may facilitate selective autophagy-associated clearance processes. In the hepatic context of inflammation, previous evidence has shown that autophagy can regulate inflammasome activation by facilitating the degradation of inflammasome-related components such as NLRP3 in liver immune cells (Kupffer cells) [34]. This supports the broader concept that autophagy may contribute to the regulation of inflammatory responses during liver injury. Consistent with this concept, deAND attenuated CDAHFD-induced inflammasome-associated caspase-1 activation in the present study. CDAHFD also induced apoptosis, as indicated by increased p17 caspase-3 expression, whereas this effect was attenuated by deAND co-treatment (Figure 7D). In agreement with these observations, a study using HepG2 cells demonstrated that PA promotes apoptosis, while preservation of normal autophagic activity exerts a protective effect against PA-induced cell death [35]. However, ROS production, autophagic flux, and mitophagic flux were not directly assessed in liver tissue. Therefore, the proposed mechanistic relationship among oxidative stress, autophagy/mitophagy, and apoptosis is primarily supported by the AML12 cell experiments, whereas the in vivo findings provide complementary evidence of hepatoprotection and changes in autophagy- and apoptosis-associated markers. Although the CDAHFD-fed mice exhibited hepatic steatosis, inflammatory cell infiltration, and fibrosis, standardized histological scoring of steatosis, lobular inflammation, and hepatocellular ballooning was not performed. Therefore, the histological findings should be interpreted as evidence of a steatohepatitis-like phenotype rather than a comprehensive NAS-based assessment of NASH severity. In addition, although serum AST was assessed as an indicator of hepatocellular injury, a broader panel of clinically relevant biochemical and metabolic markers (e.g., ALT, alkaline phosphatase, and serum triglycerides) was not comprehensively evaluated in the present study. This limitation restricts the translational interpretation of the observed hepatoprotective effects.

Beyond hepatocyte-intrinsic mechanisms, dysregulated autophagy may also interact with systemic immunometabolic pathways during NASH progression. Emerging evidence highlights the spleen–liver axis as a bidirectional immunometabolic circuit through which hepatic inflammatory signals may influence splenic immune activation, while splenic immune responses may, in turn, contribute to hepatic inflammation and disease progression [36,37]. Such inter-organ communication may provide an additional link between immune dysregulation, cellular stress, and impaired autophagic homeostasis in NASH [36,38]. Although the spleen–liver axis was not directly investigated in the present study, this emerging concept provides a broader context for understanding how hepatic autophagy dysfunction may interact with systemic immune responses and warrants further investigation.

Oxidative stress is another key contributor to PA-induced hepatotoxicity. Excess lipid accumulation promotes reactive oxygen species (ROS) generation, leading to lipid peroxidation and inflammatory cytokine release (e.g., TNF-α, IL-6, IL-1β), as well as, ultimately, to hepatocellular damage and fibrosis [26]. Various phytochemicals exhibit antioxidant properties. For example, rutin reduces lipid accumulation and oxidative stress in HepG2 cells [39], epigallocatechin-3-gallate enhances antioxidant defenses and reduces apoptosis in NAFLD models [40], and black ginseng activates AMPK signaling and antioxidant enzymes to improve NAFLD in mice [41]. Similarly, deAND has been shown to suppress oxidative stress and inhibit NLRP3 inflammasome activation by upregulating Nrf2, HO-1, and GPX expression in diet-induced models [18]. Consistent with these findings, deAND significantly reduced PA-induced ROS production by enhancing antioxidant defense-related protein expression in AML12 cells through promoting the expression of HO-1, GCLM, and GPX2. Upregulation of GPX2 and GCLM is expected to strengthen the glutathione-dependent antioxidant defense system by facilitating the detoxification of H_2_O_2_ and lipid hydroperoxides [42,43]. Although deAND reduced ROS production and improved autophagic flux, the present findings do not establish a direct causal relationship between these effects. Because the effects of NAC on autophagic flux and apoptosis were not examined, the antioxidant and autophagy-modulating effects of deAND should be considered mechanistically associated rather than causally linked. A limitation of the present study is the absence of a normal-diet-plus-deAND group; therefore, the effects of deAND on hepatic autophagy, oxidative stress, and metabolic parameters under physiological conditions remain unclear. Further studies in healthy animals are needed to more fully evaluate its hepatic safety and metabolic effects.

Given the chronic nature of NASH, the efficacy and safety of prolonged deAND administration are important considerations for its potential clinical application. In our previous study using a high-fat- and high-cholesterol-diet-induced steatohepatitis model, dietary supplementation with deAND exerted sustained hepatoprotective effects during treatment periods of up to 11 weeks, including attenuation of hepatic injury, inflammatory responses, NLRP3 inflammasome activation, and oxidative stress [18]. Additional human pharmacokinetic information is available from a phase I study of Andrographis paniculata aqueous extract containing deAND as one of its major diterpenoids. Following oral administration in healthy participants, systemic exposure to deAND was demonstrated, and repeated high-dose administration was generally tolerated, although mild adverse events and changes in liver biochemical parameters were reported [44]. Because this study evaluated a botanical extract rather than purified deAND and involved only short-term exposure, these findings do not establish the long-term safety of purified deAND. From a translational perspective, the present findings should be regarded as preclinical evidence supporting the hepatoprotective potential of deAND rather than direct evidence of clinical efficacy. Further studies are required to define its pharmacokinetic profile, optimal dosing, long-term safety, and efficacy in clinically relevant models. Ultimately, well-designed clinical trials will be necessary to determine whether the beneficial effects of deAND on autophagy, oxidative stress, inflammation, and fibrosis translate into meaningful therapeutic benefits in patients with NASH.

## 4. Materials and Methods

### 4.1. Chemical Reagents

Dulbecco’s Modified Eagle Medium/Nutrient Mixture F-12 (DMEM/F-12), fetal bovine serum (FBS), 100× insulin–transferrin–selenium (ITS), and penicillin–streptomycin were purchased from Gibco (Grand Island, NY, USA). Dexamethasone (DEX), sodium bicarbonate, HEPES, fatty acid-free bovine serum albumin (BSA), 3-(4,5-dimethylthiazol-2-yl)-2,5-diphenyltetrazolium bromide (MTT), sodium palmitate, 3-methyladenine (3-MA), chloroquine (CQ), and 2′,7′-dichlorofluorescin diacetate (DCFDA) were obtained from Sigma-Aldrich (St. Louis, MO, USA). Isopropanol and sodium dodecyl sulfate (SDS) were sourced from Merck (Darmstadt, Hesse, Germany) and Invitrogen (Carlsbad, CA, USA), respectively. Tris-HCl was purchased from VWR Co. (Radnor, PA, USA).

### 4.2. Isolation and Purification of deAND

deAND was isolated from *A. paniculata* and purified via gel filtration and recrystallization, following established protocols [16]. Briefly, powdered *A. paniculata* was extracted with 95% ethanol (1:5, *w*/*v*), and the resulting filtrate was concentrated under reduced pressure using a rotary evaporator (N1000V, EYELA, Tokyo, Japan). The concentrated extract was then dried under a chemical fume hood. The dried extract was dissolved in ethyl acetate (EA) and partitioned with an equal volume of water (1:1, *v*/*v*). The resulting ethyl acetate and aqueous fractions were separated and collected. The EA fraction was then adsorbed onto silica gel (70–230 mesh) and loaded onto a silica gel column. Gradient elution was performed using n-hexane/EA (10:1 to 1:5), followed by EA and EA/methanol (10:1 to 5:1). Fractions containing deAND, which eluted with n-hexane/ethyl acetate (EA) at ratios of 1:1 to 1:2, were identified by TLC, pooled, and subjected to recrystallization via slow evaporation. The identity and purity (~97.9%) of the obtained deAND were verified by LC-MS and HPLC, respectively.

### 4.3. Cell Culture and Palmitate Preparation

AML12 mouse hepatocytes (BCRC, Hsinchu, Taiwan) were maintained in DMEM/F12 supplemented with 10% FBS, 1X ITS (10 μg/mL insulin, 5.5 μg/mL transferrin, and 5 ng/mL selenium), 40 ng/mL dexamethasone, 15 mM of HEPES, 100 U/mL penicillin, and 100 μg/mL streptomycin. Cells were incubated at 37 °C in a humidified 5% CO_2_ atmosphere with medium replacement every 24 h.

Palmitate (PA) working solution was prepared by conjugating sodium palmitate with fatty acid-free BSA. Briefly, 100 mM of sodium palmitate was dissolved in 50% ethanol at 70 °C and subsequently complexed with 10% BSA (*w*/*v*) at a 1:19 ratio to achieve a 5 mM stock solution, which was incubated at 55 °C for 30 min before use.

### 4.4. Animal Study Design

Seven-week-old male C57BL/6 mice (National Laboratory Animal Center, Taipei, Taiwan) were acclimated for one week. Mice were housed in a temperature-controlled room (22 ± 2 °C) under a 12 h light/dark cycle with free access to food and water. Animals were monitored daily for health status and signs of distress. No unexpected adverse events occurred during the study. Mice were randomly assigned into four groups (n = 6 per group): (1) normal diet (ND; #D12450B, Research Diets, New Brunswick, NJ, USA); (2) CDAHFD (#A06071302, Research Diets); (3) CDAHFD + 0.025% deAND; and (4) CDAHFD + 0.05% deAND. deAND was mixed into the CDAHFD at concentrations of 0.025% and 0.05% *w*/*w*. Average food intake in the CDAHFD-fed groups was approximately 2.6 g/mouse/day. Based on the content of deAND in diets and average food intake, the estimated daily deAND exposure was approximately 31 and 60 mg/kg body weight/day for the 0.025% and 0.05% deAND groups, respectively. After 6 weeks of dietary intervention, mice were fasted overnight and euthanized. Livers were collected, weighed, and either fixed in 10% neutral-buffered formalin for histology or snap-frozen at −80 °C for molecular analysis. All animal protocols were approved by the China Medical University IACUC (CMUIACUC-2022-161, approved: 30 December 2021).

### 4.5. Cell Viability and ROS Measurement

Cell viability was quantified via the MTT assay. AML12 cells were seeded in 12-well plates at 0.2 × 10^6^ cells/well and were pretreated with deAND (7.5 or 15 μM) for 16 h, followed by 250 μM of PA exposure for 24 h. Formazan crystals were dissolved in isopropanol, and absorbance was measured at 595 nm (Bio-Tek Synergy HT, Winooski, VT, USA).

Intracellular ROS production was detected using the DCFDA probe. Following treatment, cells were incubated with 10 μM of DCFDA for 30 min at 37 °C. Fluorescence images were acquired using a Leica DMi8 fluorescence microscope under identical exposure settings and quantified using ImageJ software (version 1.54, National Institutes of Health, Bethesda, MD, USA). tBHP was used as a positive control.

### 4.6. Autophagic Flux Analysis

To monitor autophagic flux, cells were transfected with an RFP-GFP-LC3B plasmid for 24 h. Following deAND and PA treatment, cells were fixed and mounted with DAPI. Autophagosomes (yellow puncta: GFP^+^/RFP^+^) and autolysosomes (red puncta: GFP^−^/RFP^+^) were visualized by fluorescence microscopy. CQ was used as a lysosomal inhibitor to confirm flux impairment. For fluorescence-based autophagic flux assays, CQ was applied at 30 μM to block lysosomal degradation and verify changes in autophagic flux. In Western blot experiments involving co-treatment with PA and autophagy inhibitors, CQ was used at 1 μM to minimize excessive cytotoxicity resulting from the combined effects of PA-induced lipotoxicity and lysosomal inhibition.

### 4.7. Western Blotting

Total protein was extracted using RIPA lysis buffer and quantified via the Bio-Rad Protein Assay. Equal protein amounts were resolved by SDS-PAGE and transferred onto PVDF membranes. After blocking with 5% non-fat milk, membranes were probed with primary antibodies overnight at 4 °C, followed by HRP-conjugated secondary antibodies. Immunoreactive bands were detected using the Fuji Film LAS-4000 and quantified via ImageJ. The primary antibodies targeting the following proteins were utilized, including p-AMPK (Thr172) (Cat# 2535, Cell Signaling Technology, Danvers, MA, USA; 1:1000), Atg5 (Cat# 12994, Cell Signaling Technology, Danvers, MA, USA; 1:1000), Atg7 (Cat# 8558, Cell Signaling Technology, Danvers, MA, USA; 1:1000), LC3A/B (Cat# 12741, Cell Signaling Technology, Danvers, MA, USA; 1:1000), p-p62 (Ser403) (Cat# 39786, Cell Signaling Technology, Danvers, MA, USA; 1:1000), SQSTM1/p62 (Cat# 23214, Cell Signaling Technology, Danvers, MA, USA; 1:1000), caspase-3 (Cat# 9662, Cell Signaling Technology, Danvers, MA, USA; 1:1000), and cleaved caspase-3 (Cat# 9664, Cell Signaling Technology, Danvers, MA, USA; 1:1000); Parkin (Cat# Sc-32282, Santa Cruz Biotechnology, Dallas, TX, USA; 1:100), PINK1 (Cat# Sc-517353, Santa Cruz Biotechnology, Dallas, TX, USA; 1:200), and glutamate–cysteine ligase modifier subunit (GCLM) (Cat# Sc-22754, Santa Cruz Biotechnology, Dallas, TX, USA; 1:500); β-actin (Cat# GTX109639, GeneTex, Hsinchu, Taiwan; 1:1000) and glutathione peroxidase 2 (GPX2) (Cat# GTX100292, GeneTex, Hsinchu, Taiwan; 1:1000); glutamate–cysteine ligase catalytic subunit (GCLC) (Cat# ab41463, Abcam, Cambridge, MA, USA; 1:1000); and heme oxygenase-1 (HO-1) (Cat# 374090, Merck, Burlington, MA, USA; 1:1000).

### 4.8. Statistical Analysis

Statistical evaluations were performed using one-way ANOVA followed by Duncan’s multiple range test (SAS software version 9.4, SAS Institute Inc., Cary, NC, USA). Data are presented as mean ± SD. A *p*-value < 0.05 was considered statistically significant. For the in vivo experiments, each dietary group comprised six mice (n = 6 per group), whereas the in vitro data were derived from three or five independent experiments, as indicated in the corresponding figure legends. ANOVA was applied under the assumption of approximately normally distributed data with comparable variances across groups. We acknowledge that the reliability of formal tests of normality and homogeneity of variance is limited at these small group sizes and that this represents a limitation of the statistical approach.

## 5. Conclusions

**Figure 8:**
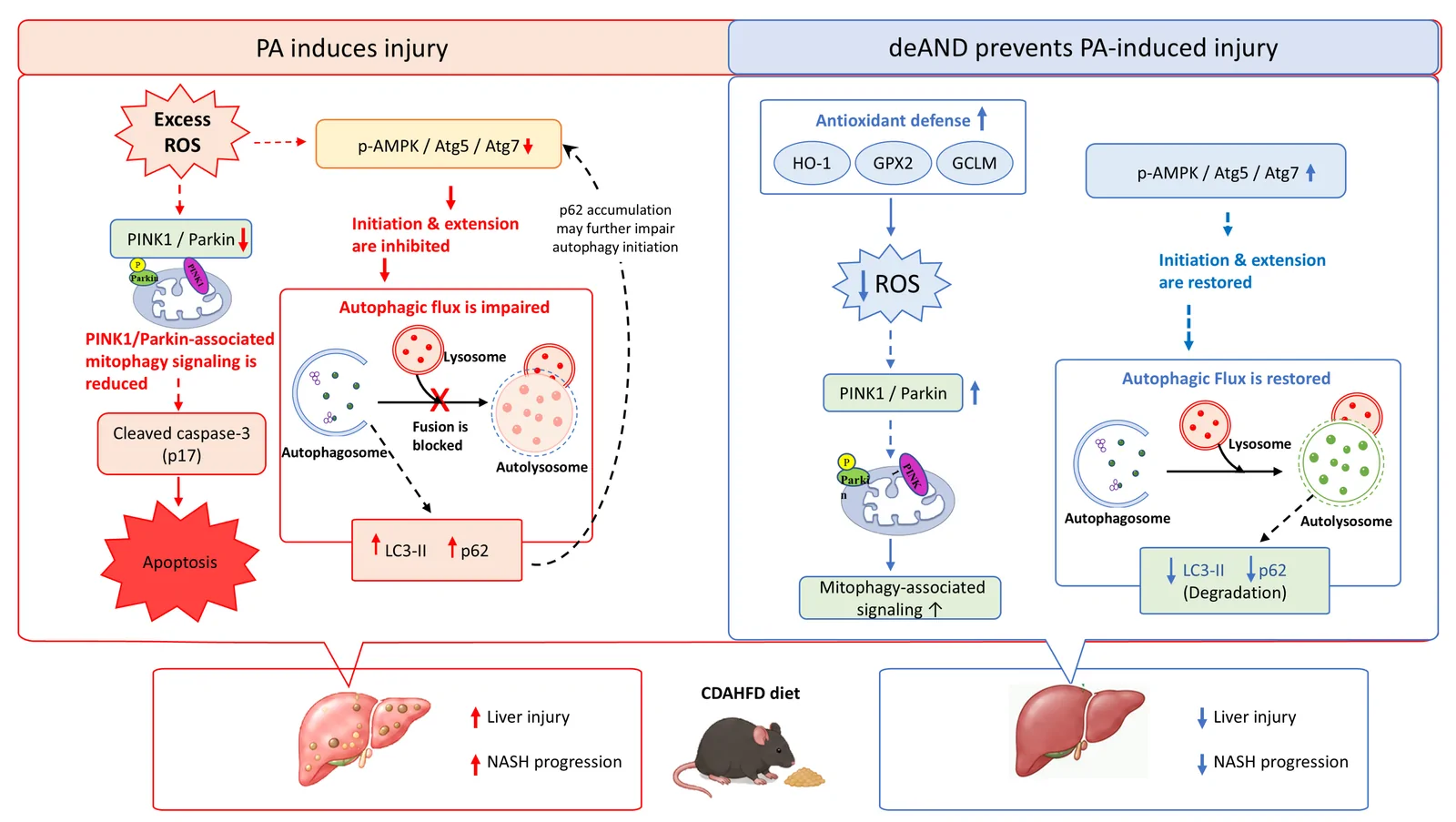
Mechanisms by which 14-deoxy-11,12-didehydroandrographolide mitigates PA-induced hepatic injury and CDAHFD-induced liver fibrosis. Upward and downward arrows indicate increased and decreased expression or activity, respectively. Solid arrows indicate experimentally supported regulatory relationships, whereas dashed arrows indicate proposed or associative relationships. A red “X” indicates inhibition or blockade of the indicated process.

In summary, deAND prevented AML12 cells from undergoing PA-induced lipotoxic stress by reducing oxidative stress, improving autophagic flux, and attenuating apoptosis. In CDAHFD-fed mice, deAND exerted hepatoprotective effects by reducing steatohepatitis, fibrosis, and apoptosis, accompanied by changes in autophagy-associated markers. The mechanistic relationships among oxidative stress, autophagy, mitophagy, and apoptosis remain to be fully established. Collectively, these findings support further investigation of deAND as a potential strategy for preventing or attenuating diet-induced metabolic liver injury. The proposed mechanisms underlying the preventive effects of deAND against PA-induced hepatic injury, primarily based on the AML12 cell experiments, together with the supportive in vivo findings, are summarized in Figure 8.

## Data Availability

The original contributions presented in this study are included in the article. Further inquiries can be directed to the corresponding author.

## Abbreviations

The following abbreviations are used in this manuscript:

AND	andrographolide
CDAHFD	choline-deficient, L-amino acid-defined, high-fat diet
CQ	chloroquine
DCFDA	2′,7′-dichlorofluorescin diacetate
deAND	14-deoxy-11,12-didehydroandrographolide
DEX	dexamethasone
DMEM/F-12	Dulbecco’s Modified Eagle Medium/Nutrient Mixture F-12
ER	endoplasmic reticulum
FBS	fetal bovine serum
FFAs	free fatty acids
GCLC	glutamate–cysteine ligase catalytic subunit
GCLM	glutamate–cysteine ligase modifier subunit
GPX	glutathione peroxidase
HCC	hepatocellular carcinoma
HO-1	heme oxygenase-1
ITS	insulin–transferrin–selenium
3-MA	3-methyladenine
MTT	3-(4,5-dimethylthiazol-2-yl)-2,5-diphenyltetrazolium bromide
NAC	N-acetylcysteine
NAFL	non-alcoholic fatty liver
NAFLD	non-alcoholic fatty liver disease
NASH	non-alcoholic steatohepatitis
ND	normal diet
PA	palmitic acid
ROS	reactive oxygen species
tBHP	tert-butyl hydroperoxide
